# Is DNA methylation the new guardian of the genome?

**DOI:** 10.1186/s13039-017-0314-8

**Published:** 2017-04-04

**Authors:** Robert M. Hoffman

**Affiliations:** 1 Anti Cancer Inc, 7917 Ostrow Street, San Diego, 92111 CA USA; 2 Department of Surgery, University of California, San Diego, CA USA

**Keywords:** Methionine, Methionine dependence, Unbalanced transmethylation, Global DNA hypomethylation, Chromosome instability, Aneuploidy, Cancer speciation

## Abstract

**Background:**

It has been known for more than 100 years that aneuploidy is an essence of cancer. The question is what keeps the genome stable, thereby preventing aneuploidy. For the past 25 years, it has been proposed that p53 is the “guardian of the genome.” However, it has been shown that inactivation of p53 does not cause aneuploidy. Another essence of cancer is global DNA hypomethylation, which causes destabilization of the genome and subsequent aneupoloidy. Yet, another essence of cancer is excessive use of methionine, resulting in methionine dependence. Methionine dependence is due to possible “metabolic reprogramming” due to carcinogens, including chemical agents and infectious organisms, such as Helicobacter pylori, that result in altered and excessive transmethylation in cancer cells. Cancer cells appear to have a “methyl-sink” whereby methyl groups are diverted from DNA.

**Conclusion:**

DNA hypomethylation destabilizes the genome, leading to aneuploidy and subsequent selection and speciation into autonomous cancers, leading to the conclusion that DNA methylation is the “guardian of the genome.”

**Electronic supplementary material:**

The online version of this article (doi:10.1186/s13039-017-0314-8) contains supplementary material, which is available to authorized users.

*“*E pur si muove*” Galileo*

## Is p53 “the guardian of the genome”?

Since Lane designated p53 as the “guardian of the genome” [1], this has become the dominant paradigm [2–4].

## DNA hypomethylation in cancer

It has been long known that global DNA hypomethylation is a general characteristic of cancer, as originally discovered by our laboratory [5, 6]. DNA hypomethylation is an early event in the formation of cancer as it appears in pre-malignant tissue [7]. The progression of DNA hypomethylation may be driven by “oncogenes” [8].

Hints regarding the effect of DNA hypomethylation on genome integrity come from Perucho et al. [9] on the relationship of *Helicobacter pylori* to gastric cancer. *Helicobacter pylori* infection is a very important risk factor for gastric cancer. Perucho et al. [9] observed that *H. pylori* appears to cause a field defect of DNA hypomethylation in a region of the gastric mucosa prior to cancer development that is not reversed by *H. pylori* eradication. Perucho et al. [9] also observed that enhanced DNA hypomethylation, was associated with a more invasive and advanced stage type of gastric cancer.

Other studies have indicated that the greater extent of DNA hypomethylation, the more malignant is the cancer. The total levels of DNA 5-methylcytosine were determined in a series of highly metastatic cell lines which had been isolated from a poorly metastatic human melanoma tumor line, MeWo, by Kerbel’s group [10]. Similar to what was observed in gastric cancer described above, the more metastatic the melanoma sublines were, the more extensively they were hypomethylated [10].

It has also been shown that global DNA hypomethylation increases as cervical dysplasia progresses to cervical carcinoma [11].

Compared to benign tumors, atypical and malignant meningiomas were observed to have increased global DNA hypomethylation [12].

## DNA hypomethylation leads to aneuploidy

Jaenish et al. generated mice with a mutant DNA methyltransferase 1 (DNMT1) allele. The mutation reduces Dnmt1 expression to 10% of normal levels and results in substantial genome-wide hypomethylation in all tested tissues. The mutant mice developed T cell lymphomas which had a high frequency of chromosome 15 trisomy. DNMT-deficient HCT-116 colon cancer cells had a high degree of genome instability leading to aneuploidy, including many novel chromosomal translocations [13]. These results indicate that DNA hypomethylation plays a causal role in chromosomal instability, aneuploidy, and subsequent cancer [14].

## Methionine

Methionine [15] is an essential amonio acid; however, it can be synthesized from homocysteine [16] and methyl-tetrahydrofolate [17, 18] in a vitamin B12-dependent reaction. Methionine is a component of proteins. Methionine is also the universal methyl donor via its activated form S-adenosylmethionine [19].

## Deprivation of methionine leads to cancer

Copeland and Salmon observed the development of liver, lung, and other cancers in a significant percentage of rats on a diet deficient in choline, which is a precursor of methionine [20]. This was the first solid evidence that methionine is involved in cancer [21].

Subsequently, Ghoshal and Farber observed that Fischer 344 male rats fed a choline-methionine deficient diet for 1–2 years during which the rats developed a 100% incidence of preneoplastic hepatocyte nodules. Subsequently, 51% of the rats developed hepatocellular carcinoma. Supplement of the diet with 0.8% choline chloride prevented the development of both the precancerous nodules and subsequent cancer [22].

The cancers resulting from methionine/choline-deprived diets in the rats were probably due to methyl shortage and subsequent DNA hypomethylation, since methionine is the source of methyl groups for DNA methylation. DNA hypomethylation appears to be an early event in choline/methionine-deficient diets, irreversible [23–26] and mediated by a deficiency in S-adenosylmethionine [27].

## Methionine dependence and altered transmethylation in cancer

The appearance of cancer in rats on choline- and methionine-deficient diets gave an early hint that perturbed methionine metabolism is involved in cancer. Sugimura noted almost 60 years ago that rat tumor growth was slowed by giving the rats a defined diet depleted in methionine for a short period of time [28]. Approximately 45 years ago, it was observed that mouse leukemia cells in culture required very high levels of methionine in order to proliferate [29]. Subsequently, most cancer cell lines were found to be methionine dependent [30, 31]. These cell lines were derived from various cancer types including liver, pancreatic ovarian, submaxillary, brain, lung, bladder, prostate, breast, kidney, cervical, colon, fibrosarcoma, osteosarcoma, rhabdomyosarcoma, leiomyosarcoma, neuroblastoma, glioblastoma and melanoma [30, 31]. Normal unestablished cell strains, thus far characterized, grow well in methionine-depleted medium [30, 32]. Many of the cancer cell lines tested have little else in common other than the fact that they are methionine-dependent and have altered methionine metabolism [33]. Human patient tumors, including tumors of the colon, breast, ovary, prostate, and a melanoma, were also found to be methionine dependent in Gelfoam^®^ histoculture [34]. The high frequency of occurrence of methionine dependence in diverse types of human cancer cells indicated that methionine dependence could be an important step in oncogenic transformation.

Three lines of evidence indicated that methionine-dependent cancer cells synthesize large amounts of methionine endogenously through the reaction catalyzed by *N* ^5^-methyltetrahydropteroyl-l-glutamate: l-homocysteine S-methyltransferase (EC 2.1.1.13) [35].

- The activity of the methyltransferase involved in methionine biosynthesis was comparable in extracts of malignant and normal cells [35].
- The uptake of radioactive label from [5-^14^C] methyltetrahydropteroyl-l-glutamic acid (*N* ^5^-methyl-H_4_PteGlu), the methyl donor for methionine biosynthesis, was at least as great in the malignant cells as in the normal cells and was nearly totally dependent on the addition of homocysteine, the methyl acceptor in methionine biosynthesis [33, 35].
- The majority of the labeled methyl groups incorporated by cancer cells was recovered as methionine [35].

We subsequently observed that although methionine-dependent cancer cells synthesized a normal amount of methionine, the level of free methionine and S-adenosylmethionine (AdoMET), which is synthesized from methionine and is the universal methyl donor, were very low in cancer cells in methionine (MET)-depleted homocysteine (HCY)-supplemented medium (MET^−^HCY^+^). In contrast, exogenously supplied MET resulted in normal levels of AdoMET. The ratio of AdoMET to S-adenosylhomocysteine (AdoHCY) is low in methionine-dependent cancer cells growing in MET^−^HCY^+^ medium, but is normal in MET^+^HCY^−^ medium. We determined that the low AdoMET/AdoHCY ratio probably limits growth of MET-dependent cells in MET^−^HCY^+^ medium [36, 37].

**Fig. 1:**
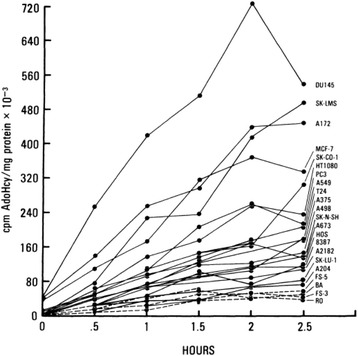
Recombinant methioninase (rMETase) traps cancer cells in S/G_2_ phase. Time-course imaging of HeLa-FUCCI cells treated with rMETase (1.0 unit/ml). Kinetics of rMETase trapping of cells in S/G_2_. Images were acquired with the FV1000 confocal microscope (Olympus, Tokyo, Japan). In the FUCCI system, the cells in G_0_/G_1_, S, or G_2_/M phases appear *red*, *yellow*, or *green*, respectively [66]

We subsequently observed that cancer cells have enhanced overall rates of transmethylation compared to normal human fibroblasts (Fig. 1) [38]. Transmethylation rates were measured by blocking AdoHCY hydrolase and measuring AdoHCY, which accumulates as a result of transmethylation from AdoMET. The excess methionine used for the enhanced transmethylation rates appears to be the basis of the methionine dependence of cancer cells which explains the low levels of free methionine and the low AdoMET/AdoHCY ratio in cancer cells under methionine deprivation [37, 38]. The elevated methionine use in cancer cells has been termed the “Hoffman effect” [39], analogous to the “Warburg effect” for enhanced glucose utilization in cancer [40]. The alteration of such a fundamental process as transmethylation in cancer may be indicative of its importance in the oncogenic process [32].

The abnormal elevated transmethylation and subsequent depletion of AdoMET appears to result in hypomethylation of DNA in cancer cells, since the methionine methyl group may be diverted to a methyl sink in the cancer cells and not sufficiently available for normal DNA methylation. For example, when the W-256 cancer cell line was cultured for 24 h in MET^−^HCY^+^ medium, the extent of methylation of nucleic acids and the acid-soluble pool of methionine were decreased. However, there was increased methylation activity of both an endogenous substrate and *Escherichia coli* tRNA. Methionine deprivation of the W-256 cells resulted in a large increase in the Vmax value for methylation of tRNA, without any change in the Km value for AdoMET [41]. These results indicated that methyl groups originating from methionine may be diverted from DNA in cancer cells.

Rare cells from methionine-dependent cancer cell lines regained the normal ability to grow in MET^−^HCY^+^ medium. These cell lines were termed methionine-independent revertants. Two revertants isolated from SV40-transformed cells had regained the ability to grow similar to normal cells in MET^−^HCY^+^ medium without substantial changes in methionine biosynthesis activity. Increased methionine biosynthesis thus is not a prerequisite to reversion from methionine dependence to independence [42, 43].

Methionine-independent revertants also had much lower basal transmethylation rates than parental methionine-dependent cell lines. For example, when comparing the methionine-dependent parent SP1 cell line and its methionine-independent revertant, the revertant SP1-R reduced its transmethylation rate. These results further suggested that methionine dependence is due to an increase in the rate of transmethylation reactions [44].

We then demonstrated that methionine-independent revertants of cancer cells concomitantly revert for characteristics associated with cancer. Of the 13 cancer-cell methionine-independent revertants characterized, 5 showed increased anchorage dependence for growth as reflected by reduced cloning efficiencies in methylcellulose, 8 showed an increased serum requirement for optimal growth, 8 showed decreased cell density in medium containing high serum, and 3 altered their cell morphology significantly. Thus, the methionine-independent revertants become more normal-like. Eight of the 13 had increased chromosome numbers, which probably played a role in reversion. Thus by selecting for methionine independence, it is possible to select for heterogeneous malignant-transformation revertants, which became less malignant, indicating further a relationship between altered methionine metabolism and oncogenic transformation [43].

**Fig. 2:**
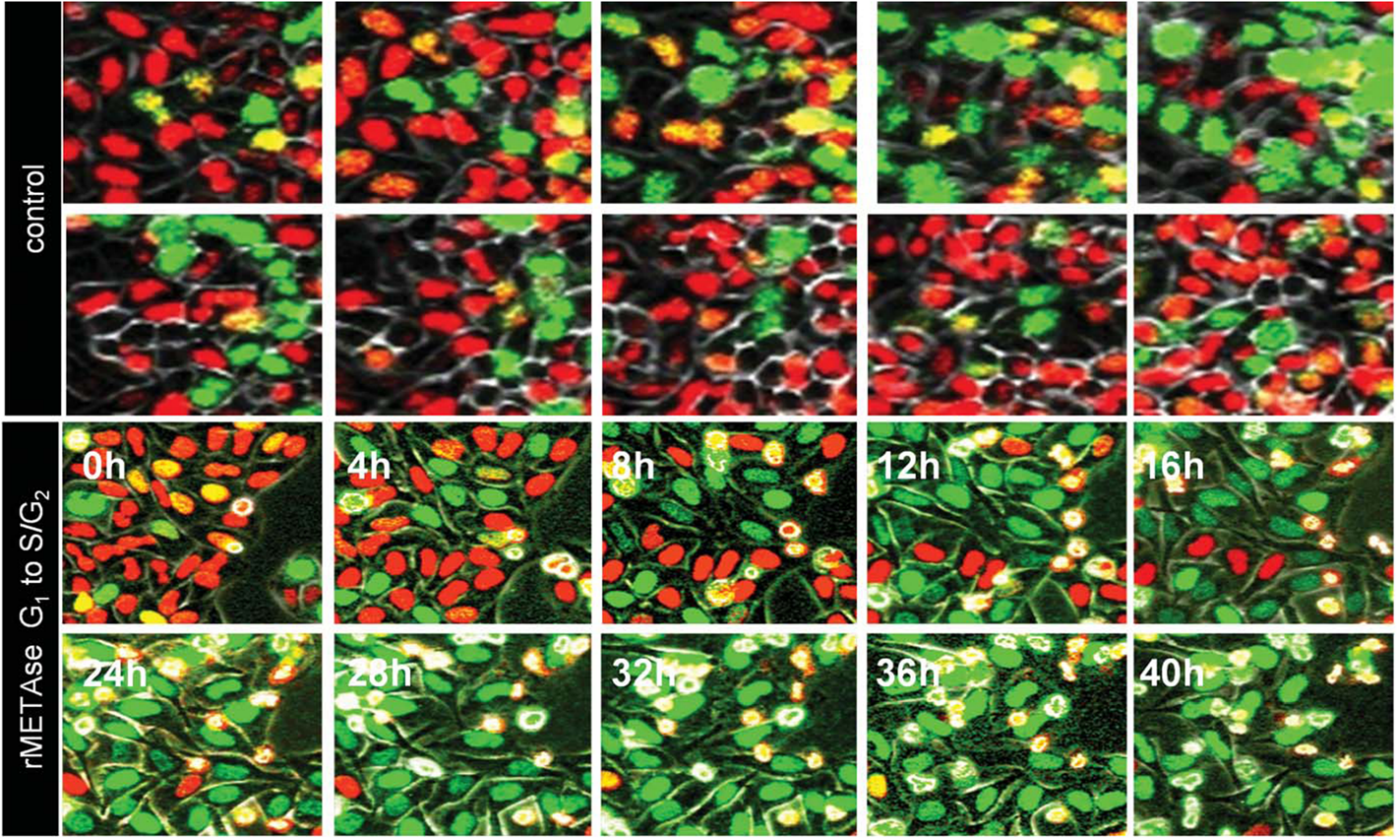
Efficacy of recombinant methioninase (rMETase)  on growth of human colon tumors HCT 15 in nude mice. rMETase (5 or 10 units/g every 8 h) was administered by i.p. injection in nude mice with human colon tumor HCT 15, growing s.c. [54]

Growth arrest of methionine-dependent cancer cells in MET ^−^HCY^+^ medium resulted in a reduction in the percentage of mitotic cells in the cell population. Fluorescence-activated cell cycle analysis demonstrated that the cells are arrested in the S/G_2_ phases of the cell cycle in MET ^−^HCY^+^ medium. This is in contrast to a G_1_-phase accumulation of cells, which occurs only in methionine-supplemented medium at very high cell densities and is similar to the G_1_ block seen in cultures of normal fibroblasts at high density [45]. Depletion of methionine in vivo by recombinant methioninase (rMETase) also resulted in S/G_2_ cell cycle arrest in tumors (Fig. 2) [46].

In summary, the altered methionine metabolism and transmethylation in cancer cells may be the initiating event of DNA hypomethylation and subsequent aneuploidy.

## Progression of aneuploid cells to autonomous cancer

Duesberg et al. [47] have proposed that carcinogenesis, such as that caused by SV40 virus, is due to induced aneuploidy, which destabilizes the karyotype which progressively evolves to autonomous clonal cancers.

Duesberg et al. [48] proposed that other carcinogens induce aneuploidy in cancer in a similar way. They induced carcinogenesis with nitrosourea and observed noninvasive mammary tumors after 2 or more months and invasive carcinomas after 6 or more months. As with SV40, these researchers observed that aneuploid karyotypes, with varying non-clonal hyperplasias, formed rare cancer “species” with individual clonal karyotypes, which became autonomous cancers.

## From perturbed methionine metabolism to DNA hypomethylation to a destabilized genome to aneuploidy to cancer

We suggest here that the pre-malignant DNA hypomethylation such as that observed by Perucho et al. [9] results from the unbalanced excess transmethylation which diverts methyl groups into where they are not available for normal methylation processes such as DNA methylation. The excess transmethylation rate explains the “methionine dependence” of cancer cells. Hypomethylation of DNA then results in the initiation of aneuploidy and subsequent speciation to autonomous cancer.

Thus, we propose that carcinogenesis is initiated by perturbation of methionine metabolism and transmethylation (“metabolic reprogramming”) resulting in DNA hypomethylation, thereby  destabilizing the karyotype, which sets off a chain reaction of aneuploidizations as Duesberg proposes, which generate ever more abnormal karyotypes and eventually cancer-specific combinations [49]. It is possible that the degree of methionine dependence of cancer cells, which may be influenced by their extent of methionine biosynthesis from homocysteine, may affect how much DNA hypomethylation has occurred in cancer cells.

This hypothesis of cancer generation described in this review can accommodate mutated “oncogenes,” “driver genes,” “tumor suppressor genes,” etc., that may influence the behavior of cancers. However, with possible rare exceptions, individual gene alterations do not account for the global changes resulting in methionine dependence, global DNA hypomethylation, and altered karotype evolution, all of which are the essences of cancer.

**Fig. 3:**
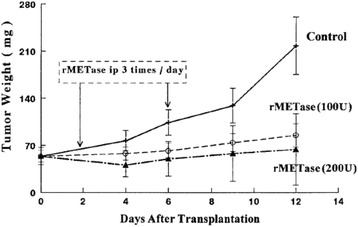
Rates of transmethylation of human tumor cell lines and normal human fibroblast cell strains. All cells were labeled with 100 μM [^35^S]-methionine-containing medium (25 μCi/ml) for 24 h. Periodate-oxidized 3-deazaadenosine was added to a concentration of 10 pM and the accumulation of [^35^S] AdoHcy was measured at half- hour intervals. *Solid lines* are human cancer cell lines. *Dashed lines* are human normal cell strains [38]

Every hypothesis should be testable and be able to be negated. The present hypothesis for example predicts that premalignant tissue  such as observed in the stomach with *H. pylori* infection or SV-40 infection, other infectious agents such as human papiloma virus, Epstein-Barr virus [50], or carcinogens such as nitrosourea or cigarette smoke, should have an elevated methionine requirement and excess and altered transmethylation leading to DNA hypomethylation as shown in the stomach after *H. pylori* infection by Perucho et al. [9]. The hypothesis predicts that chronic perturbation of methionine metabolism should be carcinogenic, such as a chronic methionine-depleted diet. The hypothesis also predicts that short-term deprivation of methionine should be curative to cancer and in mouse models; this has shown to be the case (Fig. 3) [35, 51–55]. However, important aspects of using methionine deprivation to treat cancer still remains to be investigated, including side effects on other metabolic pathways as well as the influence of the extent of methionine-synthesis capacity of cancer cells on the outcome of this therapy.

The hypothesis also explains why the use of [^11^C] methionine is so effective in positron emission tomography (PET) imaging, since the cancers use excessive methionine for their aberrant excess transmethylation and therefore take up excess [^11^C] methionine. For example, the higher specificity and sensitivity of [^11^C]-labeled methionine in PET (MET-PET) such as in gliomas has been demonstrated [56]. Because of these properties, MET-PET is the most popular amino acid tracer used in PET imaging of brain tumors and provides a high detection rate of brain tumors and metastasis and accurate tumor delineation [57]. MET-PET can also distinguish between recurrent glioma and necrosis [58]. MET-PET was heterogeneous in areas that MRI showed to be homogeneous, demonstrating the higher sensitivity of MET-PET. Malignant histopathology was detected in the areas of the tumor with the highest MET uptake, suggesting higher MET uptake correlates to a higher degree of malignancy consistent with our hypothesis [59]. MET-PET also could distinguish cures in melanoma [60].

## Conclusion

The current paradigm is that p53 is the “guardian of the genome” [1–4, 61]. However, DNA methylation may be a more global “guardian of the genome.” When DNA methylation is disrupted by aberrant (“re-programmed”) methionine metabolism, DNA becomes globally hypomethylated resulting in chromosome instability and aneuploidy leading to possible “cellular speciation” and clonal cancer [62].

It is hypothesized that carcinogens may have their initial effect by “reprogramming” methionine metabolism, in particular transmethylation. Such carcinogens include chemicals as well as infectious agents such as SV-40 virus and *H. pylori*. It is remarkable that all types of cancers tested are found to be methionine dependent [32], which is perhaps the only known general metabolic abnormality in cancer [63]. This methionine metabolism “reprogramming” results in global [5, 6] and gene-specific [64] DNA hypomethylation, another general defect in cancer, which in turn leads to aneuploidy, another general defect in cancer which in turn leads to cancer “speciation.” Vogelstein et al. [65] reported that inactivation of p53 did not lead to aneuploidy. Thus, it seems that the genome has another “guardian”, DNA methylation is a likely candidate, and when it is perturbed, the genome destabilizes leading to aneuploidy and cancer [14]. Please see (Additional file 1).

## Additional file

Additional file 1: Supplemental Material. Hoffman’s Rules for Success in Science. (DOCX 57 kb)
