# Publisher Correction: Is DNA methylation the new guardian of the genome?

**DOI:** 10.1186/s13039-018-0385-1

**Published:** 2018-06-13

**Authors:** Robert M. Hoffman

**Affiliations:** 1 AntiCancer Inc., 7917 Ostrow Street, San Diego, CA 92111 USA; 2 Department of Surgery, University of California, San Diego, CA USA

## Correction

In the original publication of this article [1] the figures and the captions of 3 figures do not match correctly due to a typographical error. In this correction article the corrected figures and captions for Figs. 1, 2 and 3 are shown.

**Fig. 1:**
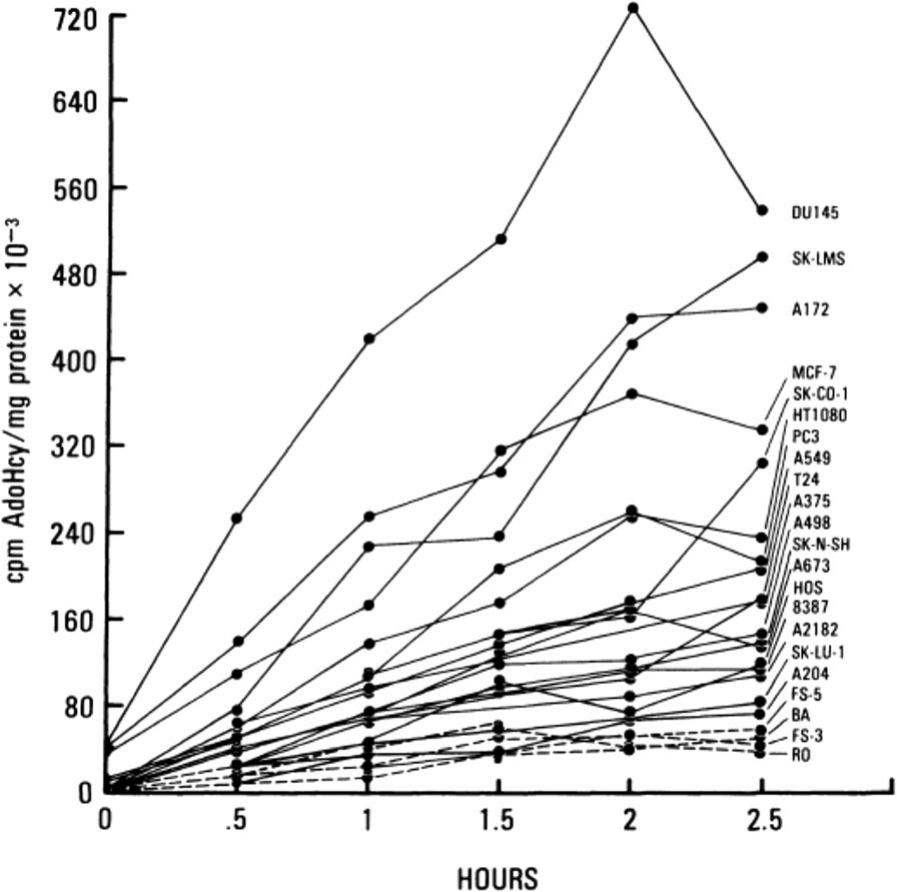
Rates of transmethylation of human tumor cell lines and normal human fibroblast cell strains. All cells were labeled with 100 μM [^35^S]-methionine-containing medium (25 μCi/ml) for 24 h. Periodateoxidized 3-deazaadenosine was added to a concentration of 10 µM and the accumulation of [^35^S] AdoHcy was measured at half- hour intervals. Solid lines are human cancer cell lines. Dashed lines are human normal cell strains [38]

**Fig. 2:**
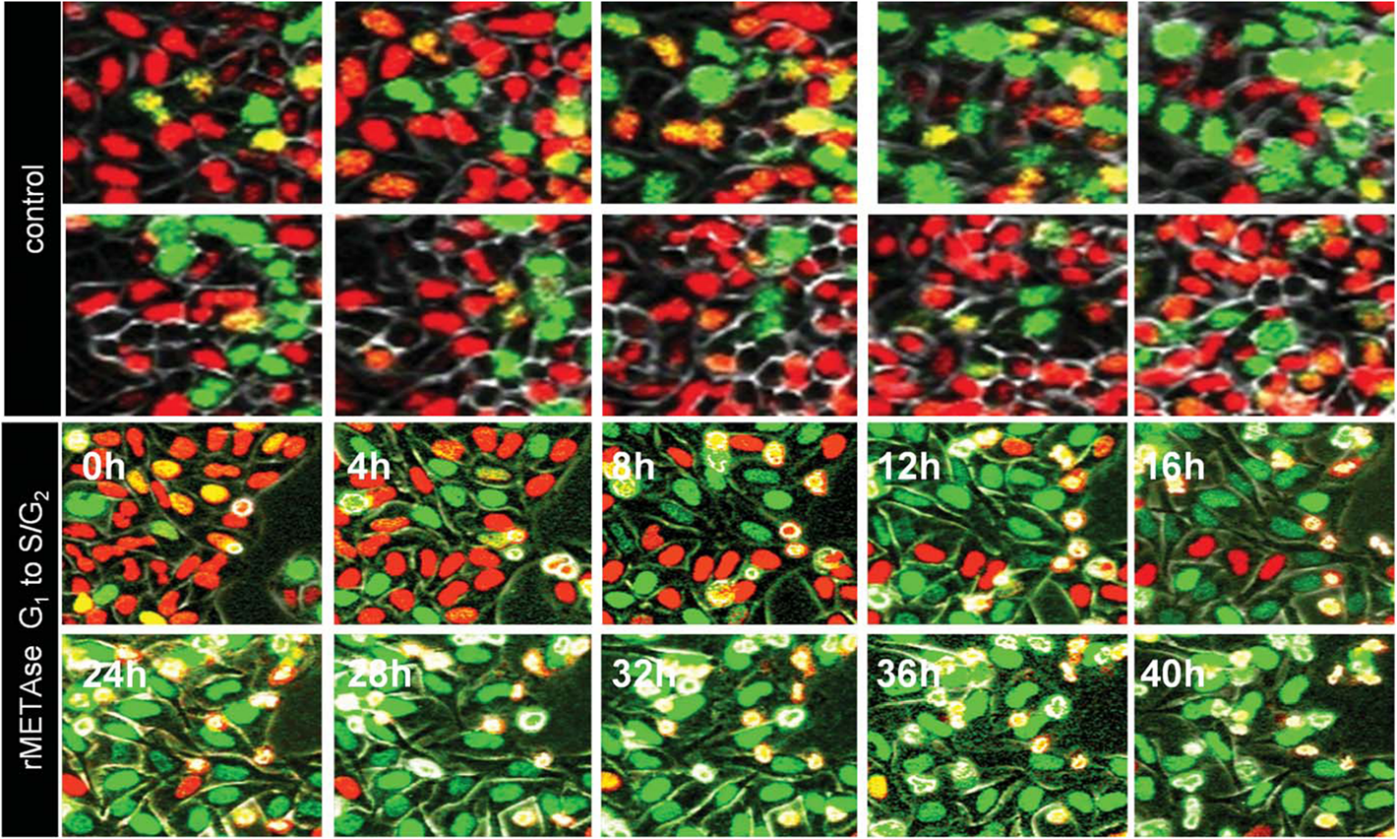
Recombinant methioninase (rMETase) traps cancer cells in S/G_2_ phase. Time-course imaging of HeLa-FUCCI cells treated with rMETase (1.0 unit/ml). Kinetics of rMETase trapping of cells in S/G_2_. Images were acquired with the FV1000 confocal microscope (Olympus, Tokyo, Japan). In the FUCCI system, the cells in G_0_/G_1_, S, or G_2_/M phases appear red, yellow, or green, respectively [66]

**Fig. 3:**
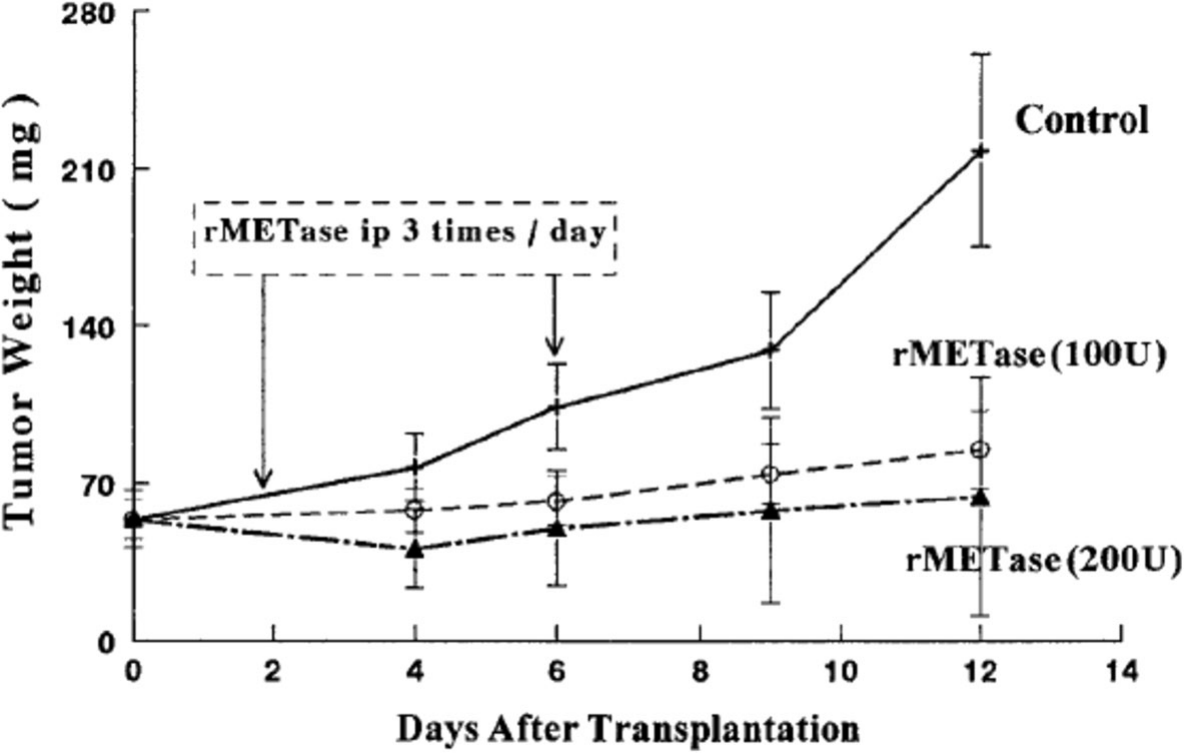
Efficacy of recombinant methioninase (rMETase) on growth of human colon tumors HCT 15 in nude mice. rMETase (5 or 10 units/g every 8 h) was administered by i.p. injection in nude mice with human colon tumor HCT 15, growing s.c. [54]

The publisher apologizes to the readers and authors for the inconvenience.
