# Supplementary material for: Is DNA methylation the new guardian of the genome?
Source: Mol Cytogenet. 2017 Apr 4;10:11. doi: 10.1186/s13039-017-0314-8 (PMC5381125; doi:10.1186/s13039-017-0314-8)
Supplement: Additional file 1: — Supplemental Material. Hoffman’s Rules for Success in Science. (DOCX 57 kb) [file 13039_2017_314_MOESM1_ESM.docx]

**-HOFFMAN’S RULES FOR SUCCESS IN SCIENCE-**

**(On a variation of a theme of A. R. Moossa’s Rules of Surgery)**

**1.** Do not become a scientist. You will be far better off selling used cars.

**2.** If you become a scientist, do not be creative since nobody will be interested in or understand your work or publish or fund it, and you will be ostracized. Your creative work will eventually be stolen, and the thief will take full credit.

**3.** If you want to try to be a successful scientist, steal other people’s work, and do not cite their papers. Fake the data if you think it helps.

**4.** Always follow “opinion leaders,” right or wrong.

**5.** Attack and ostracize anyone who is against the “opinion leaders.” Trash their manuscripts and grant applications, if you are a reviewer. Steal the data from the breakthrough manuscripts you trash and publish as fast as possible. Submit your grant applications based on the best grant applications you trashed.

**6.** Almost every scientific paper you read will be wrong, but don’t worry about it.

**7.** When you read scientific papers that are five years old, most will make you laugh, since they will seem so ridiculous.

**8.** When you read scientific papers that are 10 years old, most will make you clinically depressed.

**9.** Don’t worry if your papers get retracted. There are infinitely more papers to write.

**10.** Never trust another scientist, since he/she will do to you what you want to do to him/her: steal their work and then trash him/her.

**11.** If some scientific fashion goes out of style, don’t worry, just wait long enough and it will come back in a slightly different guise, right or wrong. Copy old seminal papers and submit them as new ones with your name as author in time for them to be back in fashion and take credit as the pioneer.

**12**. Invent a catchy term or phrase for an imaginary function of a gene.

**13.** Scientific truth doesn’t matter: only fame and fortune count.

Robert M. Hoffman, Ph.D.

Lessons from my first 50 years in science.
